# The effect of liposomal bupivacaine injection during total hip arthroplasty: a controlled cohort study

**DOI:** 10.1186/1471-2474-15-310

**Published:** 2014-09-24

**Authors:** Benjamin G Domb, Asheesh Gupta, Jon E Hammarstedt, Christine E Stake, Kinzie Sharp, John M Redmond

**Affiliations:** American Hip Institute, 1010 Executive Court Suite 250, Westmont, IL 60559 USA; Loyola University Chicago, Hinsdale Orthopaedics, American Hip Institute, 1010 Executive Court Suite 250, Westmont, IL 60559 USA; Mayo Clinic Florida, 4500 San Pablo Road, Jacksonville, FL 32224 USA

## Abstract

**Background:**

Multimodal analgesia following total hip arthroplasty has been shown to improve patient satisfaction, participation with physical therapy, and allow early return to self care. Liposomal bupivacaine is a formulation of local anesthetic which has the potential to provide anesthesia for up to 72 hours following administration. The purpose of this study was to compare the effectiveness of liposomal bupivacaine with bupivacaine following total hip arthroplasty.

**Methods:**

A retrospective chart review was performed on 28 patients undergoing total hip arthroplasty or hip resurfacing who received intraoperative administration of liposomal bupivacaine. A control group of 30 patients who had previously undergone total hip arthroplasty or hip resurfacing and had received intraoperative administration of bupivacaine also underwent a chart review. Length of stay, post-operative opioid use, and pain scores were compared for both groups.

**Results:**

The average length of stay in the study group was 1.93 days and the control group length of stay was 2.47 days (p ≤ 0.05). Morphine equivalent use was less in the study group during the first 24 hours compared to the control group (p ≤0.05). During the second and third 24 hours the morphine equivalent use difference was not statistically significant. Visual analogue scores were not significantly different between groups at any time point.

**Conclusion:**

Liposomal bupivacaine administration during total hip arthroplasty appears to decrease the need for opioid use post operatively and decrease length of stay. The results of this study justify the need for a well-designed randomized controlled trial utilizing liposomal bupivacaine as part of multimodal analgesia during THA.

**Electronic supplementary material:**

The online version of this article (doi:10.1186/1471-2474-15-310) contains supplementary material, which is available to authorized users.

## Background

Multimodal analgesia following total hip arthroplasty (THA) has been shown to improve patient satisfaction, participation with physical therapy, and allow early return to self care [1]. Opioid related side effects are dose dependent, and efforts to minimize opioid consumption through peripheral nerve blockade, epidural anesthesia, local anesthetics, and non-opioid medications have been effective [2–5]. Ideally, following THA, analgesic related side effects are minimized to allow early mobilization and recovery [6].

Epidural and peripheral nerve blockade have been used as methods to decrease post operative opiate consumption following THA [2, 7]. Epidural anesthesia is an effective method of pain control. However, muscular blockade can reduce a patient’s ability to participate in physical therapy, and the need for venous thromboembolism prophylaxis puts patients at risk for epidural hematoma. Lumbar plexus and femoral nerve blocks have been used as a single shot and continuous infusion for postoperative analgesia following THA [2, 8]. Continuous lumbar plexus blocks have been shown to be effective in reducing pain scores, opioid consumption, and side effects [2]. However, trained personal, operating room delays, and post-operative motor weakness have been barriers to routine lumbar plexus block use. The use of a catheter and potential need for monitoring are also barriers to outpatient THA.

The use of local anesthetics and local infiltration analgesia has been used following THA with mixed results [4, 5, 9, 10]. A recent level one study compared local infiltration analgesia to a placebo as part of multimodal analgesia following THA and found no benefit in the study group [9]. This study was also unable to detect a difference between patients with continuous postoperative intra-articular infusion of ropivicaine compared to patients not receiving an infusion. This contrasts with previous randomized controlled trials investigating infiltration analgesia in the setting of THA, which have shown a benefit compared to a control group, and when compared to patients receiving epidural analgesia [4, 5].

Liposomal bupivacaine is a formulation of local anesthetic approved by the FDA in October 2011 for single-dose infiltration in the surgical site for postsurgical analgesia. Bupivicaine is released from multivesicular liposomes over a period of time, which may result in local anesthetic action for up to 72 hours. This method of delivery has to the potential reduce opioid consumption without the need for a nerve block or catheter placement. To our knowledge, this method of local anesthetic delivery has not been investigated during THA. The purpose of this study was to compare the effectiveness of liposomal bupivacaine with standard bupivacaine following total hip arthroplasty. We hypothesized that narcotic use and length of stay would be decreased by the use of liposomal bupivacaine. Pain scores have historically been relatively low using narcotic pain control, and therefore were expected to remain unchanged while the use of narcotics was decreased.

## Methods

### Study design

A retrospective chart review was performed on 28 patients undergoing total hip arthroplasty or hip resurfacing who received intraoperative administration of liposomal bupivacaine. A control group of 30 patients who had previously undergone total hip arthroplasty or hip resurfacing and had received intraoperative administration of bupivacaine also underwent a chart review. The study period was from November 2012 to May 2013. Patients received a preoperative analgesic regimen, which typically included 1000 mg of oral acetaminophen, 400 mg of oral celecoxib, 75 mg of oral pregabalin, and 10 mg of oral extended release oxycodone. Exclusion criteria were revision surgery, previous opioid dependence, and surgical approaches other than posterior.

### Surgical technique

All surgical procedures were performed by the senior surgeon (BGD). All patients had total hip arthroplasty or hip resurfacing through a posterior approach. During wound closure all patients underwent injection of liposomal bupivacaine or bupivacaine throughout the hip capsule, external rotators, gluteus medius, gluteus minimus, gluteus maximus, tensor fascia lata, vastus lateralis, and subcutaneous tissues. Intra-operative analgesia was used by anesthesiologists and consisted of fentanyl or hydromorphone as needed for pain control.

### Bupivicaine administration

All patients received an intra-operative local anesthetic injection. The study group received 20 mL (266 mg) of liposomal bupivacaine mixed with 40 mL of 0.25% bupivacaine with epinephrine. The control group received 60 mL of 0.25% bupivacaine with epinephrine. Care was taken to avoid the sciatic and femoral nerves during administration. Both liposomal bupivacaine and bupivacaine are considered part of the standard of care in hip arthroplasty.

### Postoperative care

All patients were allowed to discharge from the hospital when they met discharge criteria. Discharge criteria included adequate pain control utilizing an oral regimen, tolerating oral intake, and ability to self care. Nurses observed patients postoperatively and administered opioid medication as needed for comfort. Patients and nurses utilized ketorolac, hydrocodone, acetaminophen, codeine, fentanyl, hydromophone, extended release oxycodone, and oxycodone for pain control. Physical therapy was initiated within 24 hours following surgery to assist with ambulation.

### Outcome measures

Following surgery patients were discharged when they met criteria. Length of stay was recorded in days for all patients. Post operative opioid consumption was recorded for all patients in the hospital. Opioids administered were converted to morphine equivalents for all patients. Opioid use was tabulated as morphine equivalents in the first 24 hours, second 24 hours, and third 24 hours following surgery. Opioid use after 72 hours was not collected. Patients reported their pain on a visual analog scale (VAS) from 0 to 10, where 0 was considered to be no pain at all and 10 was considered to be the worst possible pain. Average pain scores were tabulated for the first 24 hours, second 24 hours, and third 24 hours following surgery. Readmissions related to pain control were recorded.

### Statistics

A chi-squared analysis was used to compare categorical data between groups such as gender distribution. The two-tailed, independent *t*-test was used to assess length of stay, morphine use, and visual analogue scores. An A-priori sample size was calculated for a two-tailed hypothesis utilizing a mean difference in morphine equivalent use of 7 mg with a standard deviation of 8 mg [11]. A sample size of 34 patients would be needed for a power (beta) of 0.8 and probability level (alpha) of 0.05. A p-value of <0.05 was considered significant. Statistical analysis was done using Microsoft Office Excel 2007 (Redmond, Washington, USA).

### Ethical approval

Institutional Review Board approval from Adventist Hinsdale Hospital, affiliated with Advocate Healthcare, was obtained and patient informed consent was obtained as well. STROBE guidelines for observational studies were adhered to for this study,

## Results

Patient demographics are displayed in Table 1. No significant differences between groups were noted for age or gender. The study group included 24 THA patients and 3 hip resurfacing patients, and the control group included 20 THA patients and 10 hip resurfacing patients. The control group did include more hip resurfacing patients than the study group (p < 0.05). One patient from the study group was excluded due to previous opioid tolerance, leaving 27 patients for evaluation.

**Table 1:** **Cohort demographic breakdown for gender, age and surgical mode between patients in the study and control groups**

	Demographics				
	Study		Control		
	Count/Average	Percentage	Count/Average	Percentage	P-Value
**Male**	11	41%	17	57%	
**Female**	16	59%	13	43%	
**Total**	27		30		0.230
**Age**	55.5		55.80		0.899
**Surgery type**					
THR	24	89%	20	67%	
Resurfacing	3	11%	10	33%	
Total	27		30		0.000

The average length of stay in the study group was 1.93 days and the control group length of stay was 2.47 days (p ≤ 0.05) (Table 2).

**Table 2:** **Study measurements between the study and control group for length of stay, pain measurements for the first 72 hours, and morphine equivalents for the first 72 hours**

	Study		Control		t-test
	Average	Count	Average	Count	
**Length of stay**	1.93	27	2.47	30	0.050
**Morphine**					
First 24 hours	24.00	27	53.35	30	0.000
Second 24 hours	41.08	13	64.94	25	0.102
Third 24 hours	39.58	6	48.77	13	0.651
**Pain scores**					
First 24 hours	2.81	27	2.82	30	0.968
Second 24 hours	3.27	12	3.29	25	0.964
Third 24 hours	2.20	7	2.61	13	0.557

**Figure 1:**
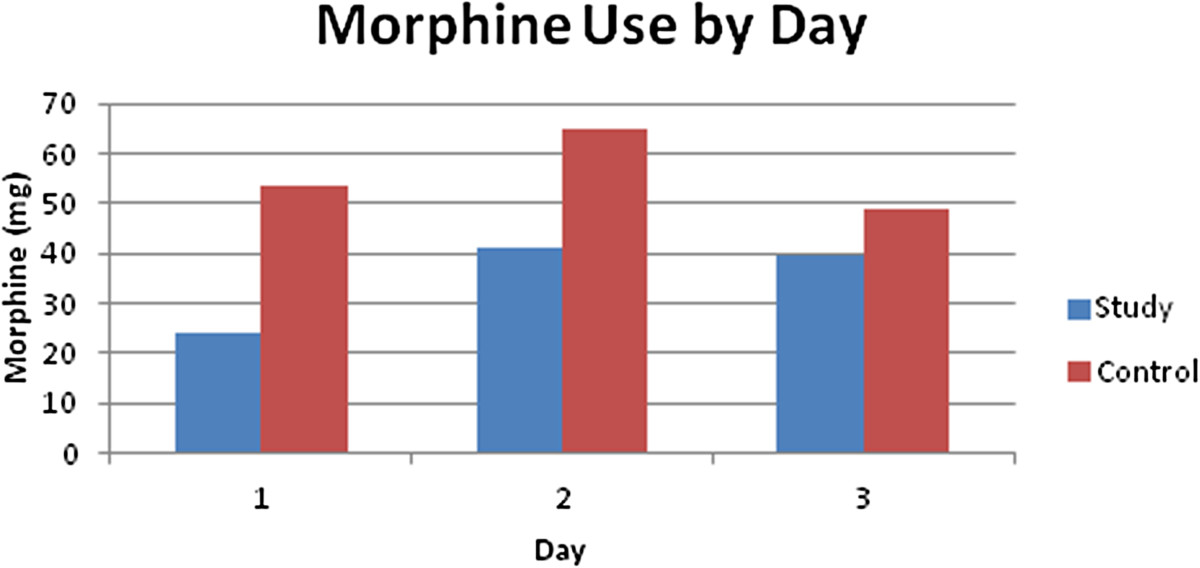
**Morphine equivalent use in milligrams during the first 24 hour period, the second 24 hour period and third 24 hour period following surgery for the study and control groups.**

The study group and control group morphine equivalent use in the first, second, and third 24 hour intervals are displayed in Table 2. Morphine equivalent use was less in the study group during the first 24 hours compared to the control group (p ≤ 0.05) (Figure 1). During the second and third 24 hours the morphine equivalent use difference was not statistically significant. Morphine equivalent use could not be calculated following hospital discharge. In the study group 13 patients were available for the second 24 hours, and 6 were available for the third 24 hours. In the control group 25 patients were available for the second 24 hours, and 13 were available for the third 24 hours.

**Figure 2:**
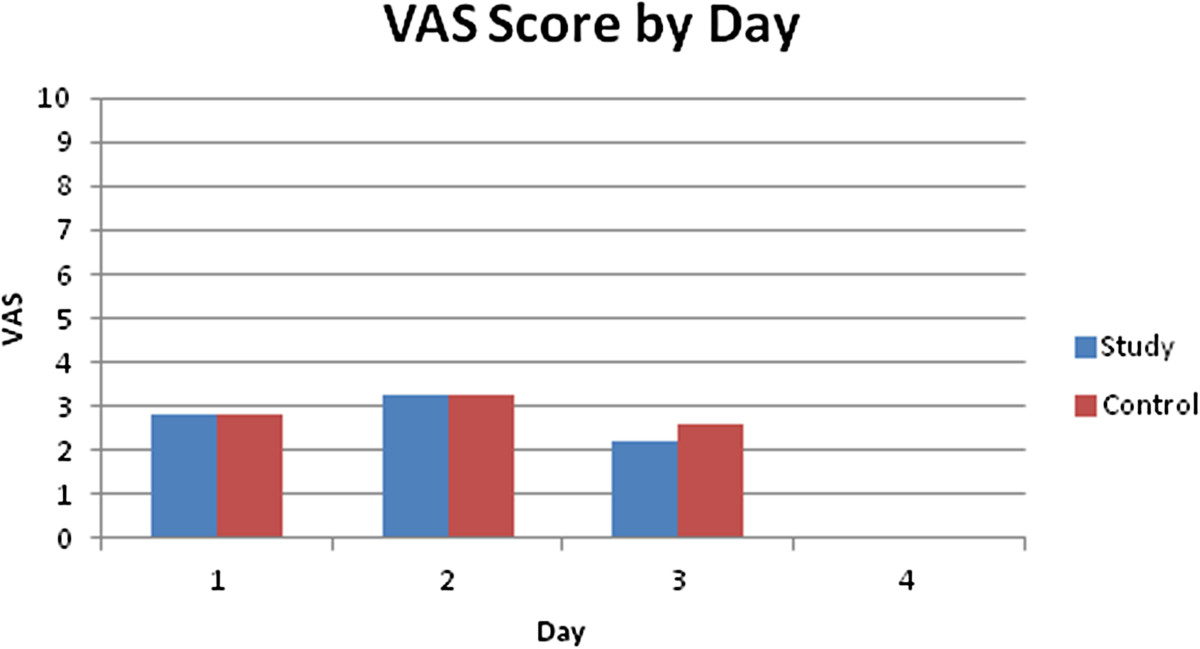
**VAS average during the first 24 hour period, the second 24 hour period and third 24 hour period following surgery for the study and control groups.**

The study group and control group average VAS during the first, second, and third 24 hour intervals are displayed in Table 2. VAS was not significantly different between groups at any time point (Figure 2). VAS could not be calculated following hospital discharge. In the study group 12 patients were available for the second 24 hours, and 7 were available for the third 24 hours. In the control group 25 patients were available for the second 24 hours, and 13 were available for the third 24 hours.

There were no readmissions secondary to pain control in either group. The one patient excluded from the study group for history of opioid use was readmitted for pain control. This patient required more morphine equivalents during the first 24 hours (1,150 mg) than the rest of the study group combined.

## Discussion

Multimodal analgesia following total hip arthroplasty (THA) has the potential to improve pain control and limit opioid related side effects. Recent efforts to use local anesthetics in THA have been limited to peripheral nerve blockade and local infiltration analgesia [1, 5, 9, 10, 12]. Liposomal bupivacaine is a novel method of local anesthetic delivery, and obviates the need for nerve blocks and catheter placement. In the present study, patients treated with liposomal bupivacaine demonstrated decreased length of stay and decreased morphine equivalent use during the first 24 hours following total hip arthroplasty, when compared to a control group treated with standard bupivicaine.

Given the recent approval of liposomal bupivacaine, there are no similar studies on the use of this delivery method for THA. The results of this pilot study will be used to design a randomized controlled trial. Bramlett et al. investigated the use of a liposomal release bupivacaine during total knee arthroplasty and noted improved analgesia compared to patients treated with bupivacaine infiltration alone [13]. The results of the current study mirror the decreased morphine equivalent use in patients treated with liposomal bupivacaine, although we did not detect a significant difference in pain scores between groups.

There have been recent reports in the general surgery literature examining the effect of liposomal bupivacaine for post operative analgesia [14–16]. To our knowledge there is one randomized controlled trial involving liposomal bupivacaine in the literature [17]. This study involved patients undergoing hemorrhoidectomy and compared liposomal bupivacaine to placebo. This trial demonstrated decreased pain, opioid requirements, delayed time to first opioid use, and improved patient satisfaction in the study group. In this study the reduction in pain lasted 72 hours. The current study differs from this randomized trial in that the control group in the current study received standard bupivacaine, not a placebo.

The current study is the first description of the use of liposomal bupivacaine for THA in the literature. It compares a similar group of patients, all of which underwent a posterior approach for hip arthroplasty by a single surgeon. It also addresses the clinically relevant question – whether liposomal bupivacaine yields improved results compared to standard bupivacaine; rather than comparing liposomal bupivacaine to a placebo or intravenous patient controlled analgesia. Finally, statistically significant differences were found in length of stay and postoperative narcotic use.

This study has many limitations. The retrospective nature of this study precludes the ability to standardize preoperative and postoperative multimodal analgesia, select patients, and standardize perioperative care, which may have led to undetectable bias between groups. However, the groups were homogeneous in that they consisted of only posterior approach hip arthroplasties, all performed by the same surgeon, thus removing surgeon technique and surgical approach as confounding variables. Second, there were more hip resurfacings in the control group. Third, the average age of arthroplasty patients in this study for both groups is 55 years; this represents a slightly younger patient cohort than many arthroplasty practices. Whether these results can be extrapolated to an older patient population is unknown.

## Conclusion

Liposomal bupivacaine administration during total hip arthroplasty appears to decrease the need for opioid use post operatively and decrease length of stay requirements. The results of this study justify the need for a well-designed randomized controlled trial utilizing liposomal bupivacaine as part of multimodal analgesia during THA.

## Authors’ original submitted files for images

Below are the links to the authors’ original submitted files for images. Authors’ original file for figure 1 Authors’ original file for figure 2
