# Supplementary figures and images for: The effect of liposomal bupivacaine injection during total hip arthroplasty: a controlled cohort study
Source: BMC Musculoskelet Disord. 2014 Sep 24;15:310. doi: 10.1186/1471-2474-15-310 (PMC4190483; doi:10.1186/1471-2474-15-310)

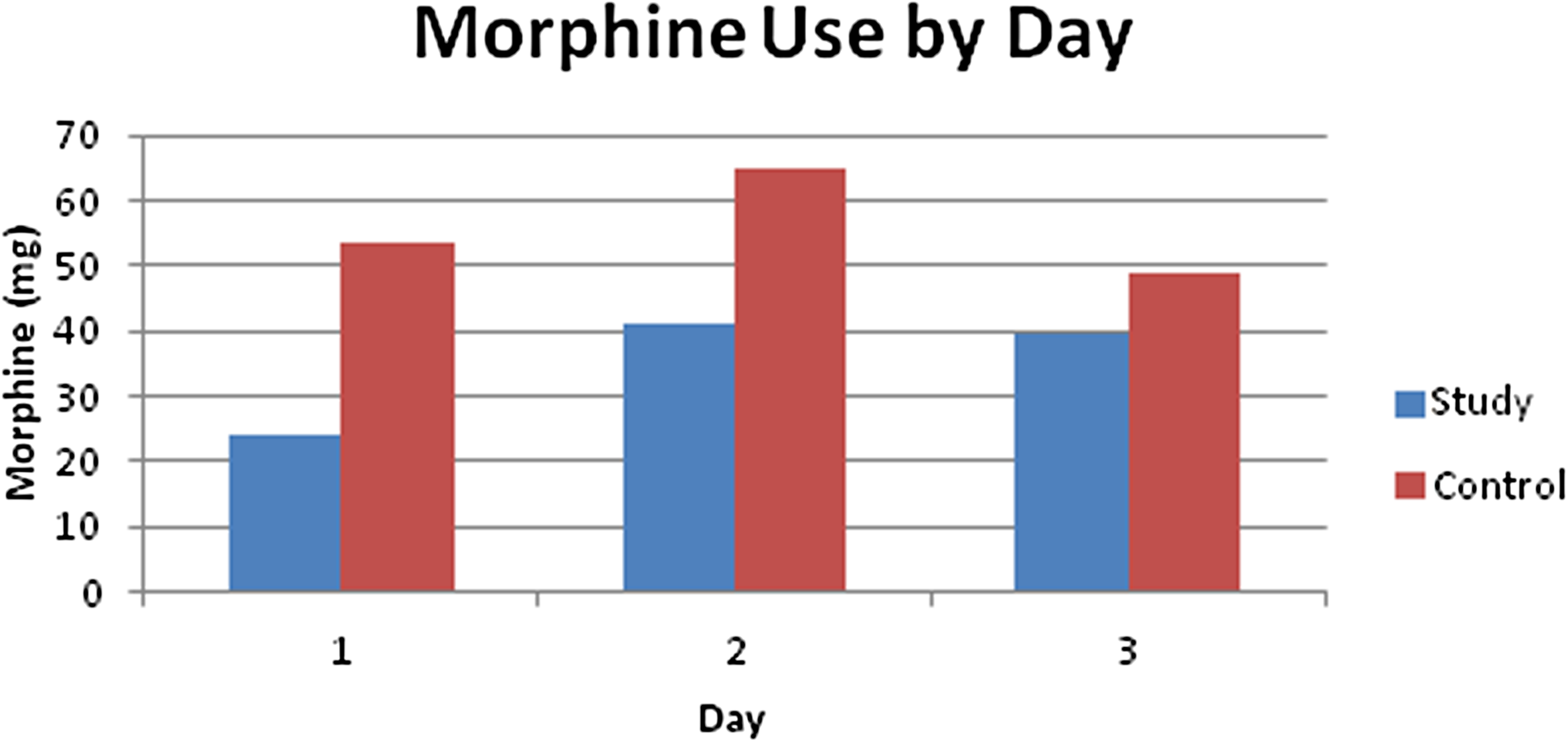

Supplement: Supplementary file 1 — Authors’ original file for figure 1 [file 12891_2014_2255_MOESM1_ESM.tif]

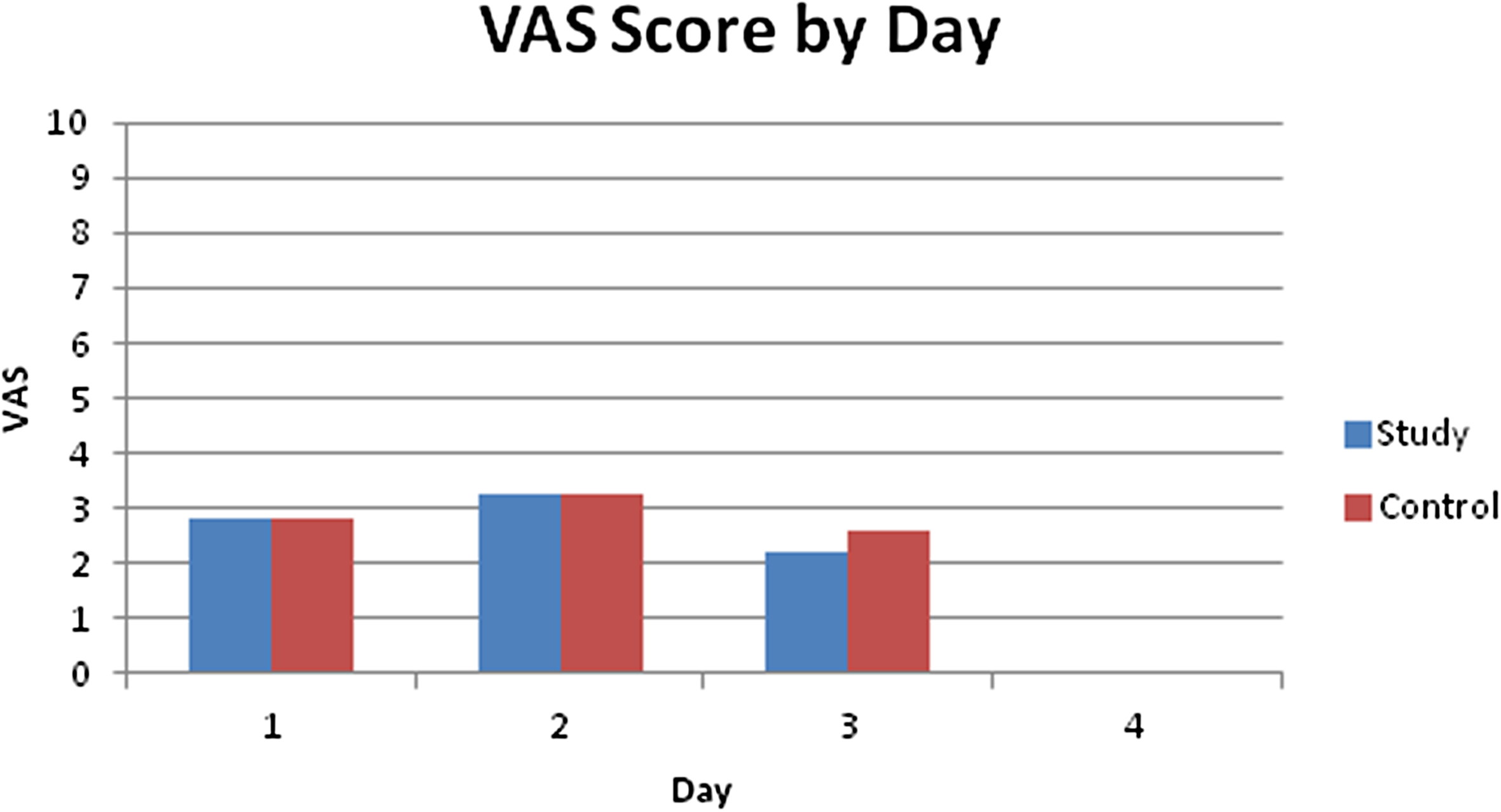

Supplement: Supplementary file 2 — Authors’ original file for figure 2 [file 12891_2014_2255_MOESM2_ESM.tif]
